# Releasing Behavior of Lipopolysaccharide from Gelatin Modulates Inflammation, Cellular Senescence, and Bone Formation in Critical-Sized Bone Defects in Rat Calvaria

**DOI:** 10.3390/ma13010095

**Published:** 2019-12-23

**Authors:** Jianxin Zhao, Yoshitomo Honda, Tomonari Tanaka, Yoshiya Hashimoto, Naoyuki Matsumoto

**Affiliations:** 1 Department of Orthodontics, Osaka Dental University, 1-5-17, Otemae, Chuo-ku, Osaka 540-0008, Japan; jianxinzhao@hotmail.com (J.Z.); naoyuki@cc.osaka-dent.ac.jp (N.M.); 2 Institute of Dental Research, Osaka Dental University, 8-1, Kuzuhahanazonocho, Hirakata, Osaka 573-1121, Japan; 3 Graduate School of Science and Technology, Kyoto Institute of Technology, Matsugasaki, Sakyo-ku, Kyoto 606-8585, Japan; t-tanaka@kit.ac.jp; 4 Department of Biomaterials, Osaka Dental University, 8-1, Kuzuhahanazonocho, Hirakata, Osaka 573-1121, Japan; yoshiya@cc.osaka-dent.ac.jp

**Keywords:** lipopolysaccharide, inflammation, cellular senescence, bone formation

## Abstract

Lipopolysaccharide (LPS) is a well-known strong inducer of inflammation. However, there is little information regarding how LPS-release behavior affects cellular senescence at the affected area. In this paper, we demonstrate that a vacuum-heating technique (dehydrothermal treatment) can be utilized to prepare an LPS sustained-release gelatin sponge (LS-G). LPS sustained release from gelatin leads to the long-term existence of senescent cells in critical-sized bone defects in rat calvaria. Three types of gelatin sponges were prepared in this study: a medical-grade gelatin sponge with extremely low LPS levels (MG), LS-G, and a LPS rapid-release gelatin sponge (LR-G). Histological (H-E) and immunohistochemical (COX-2, p16, and p21) staining were utilized to evaluate inflammatory reactions and cellular senescence one to three weeks after surgery. Soft X-ray imaging was utilized to estimate new bone formation in the defects. The LR-G led to stronger swelling and COX-2 expression in defects compared to the MG and LS-G at 1 week. Despite a small inflammatory reaction, LS-G implantation led to the long-term existence of senescent cells and hampered bone formation compared to the MG and LR-G. These results suggest that vacuum heating is a viable technique for preparing different types of materials for releasing bacterial components, which is helpful for developing disease models for elucidating cellular senescence and bone regeneration.

## 1. Introduction

Bacterial infection is a major obstacle to bone fracture healing [1,2] and bone-regeneration therapies [3]. It is a consensus that bacterial components modulate bone metabolisms. Despite the development of various antibacterial materials [4], antibiotic use occasionally exhibits poor effectiveness in terms of preventing recurrent infections [5] and implant failures [6]. Further elucidation of the mechanisms between chronic inflammation induced by residual bacterial components and bone regeneration is crucial for preparing advanced biomaterials for bone regeneration therapy. 

Lipopolysaccharide (LPS), which is a typical outer-cell membrane of Gram-negative bacterial endotoxins, is a latent contaminant in many treatments and surgeries in the medical field [7]. In general, bacterial LPS consists of three key components, namely a polysaccharide shell, oligosaccharide regions near the core, and lipid A structures in the very center [8]. LPS exhibits high heat resistance, even under typical sterilization conditions [7]. LPS is a well-known stimulant that strongly activates immune system signals and inflammation [9]. Specifically, LPS activates M1 macrophages, which play an important role in secreting inflammatory cytokines and generating reactive oxygen species [10]. Regarding bone biology, LPS stimulation promotes the formation of osteoclasts by increasing receptor activator of NF-kappaB ligand (RANKL) levels or directly stimulating osteoclast progenitor cells [11], leading to bone resorption. LPS impairs bone healing at the systemic administration level [9] while promoting osteoblast differentiation at low doses [12]. Overall, the effects of LPS on bone formation are still controversial. 

Various stresses, such as oxidative stress and inflammatory cytokines, irreversibly arrest the cell cycle, thereby generating stress-induced senescent cells both in vivo and in vitro [13]. LPS can induce DNA damage [14] and generate stress-induced primary senescent cells in vitro [15,16]. However, information regarding the LPS induction of senescent cells in vivo is still lacking. In general, biological experiments, LPS has been tested based on one-time local administration [17,18] or systemic administration [19,20,21], or based on recurrent systemic administration [22]. Few studies have analyzed the contribution of LPS release behaviors to host reactions, particularly for in vivo cellular senescence. 

It is widely recognized that the controlled release of drugs is a promising strategy for eliciting the full functionality of therapeutics [23]. Bioengineering approaches enable various materials to release drugs in a controlled manner [24]. Gelatin, denatured collagen, has been widely utilized as a substrate for biomaterials and drug carriers [25,26,27]. Various groups, including our own group, have utilized this protein as a controlled release carrier for polyphenol [28], growth factors, and antibiotics [27]. A vacuum-heating technique (dehydrothermal treatment: DHT) has been utilized to promote intermolecular bonding based on esterification between the carboxyl and hydroxyl groups in polymers [29]. Based on this background, we hypothesize that the combination of gelatin and LPS with or without DHT should enable us to prepare different LPS release materials for developing chronic inflammation models with residual LPS levels. 

**Figure 1:**
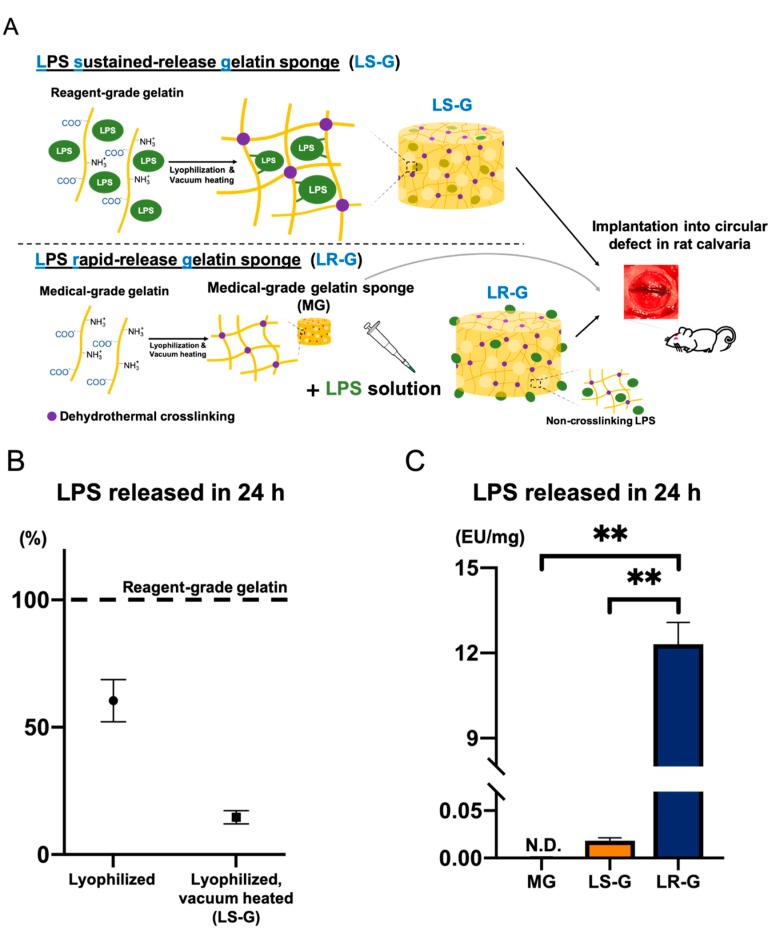
(**A**) Flow chart for sponge preparation. (**B**) Lipopolysaccharide (LPS) release from the lyophilized reagent-grade gelatin sponge or LS-G over 24 h in saline. The measurement value for reagent-grade gelatin powder is set to 100%. (**C**) LPS release from medical-grade gelatin sponge without LPS (MG), LPS sustained-release gelatin sponge (LS-G), and LPS rapid-release gelatin sponge (LR-G) into saline over 24 h. Mean with standard deviation (SD) (*n* = 3). ** *p* < 0.01: one-way analysis of variance (ANOVA), Tukey–Kramer test as post hoc test. N.D. Not detected.

In this study, we attempted to evaluate the relationships between inflammation, cellular senescence, and bone formation in early stages under different LPS release behaviors from the gelatin sponges in vivo. We prepared three different types of gelatin sponges representing distinct release behaviors of LPS: an LPS sustained-release gelatin sponge (LS-G), LPS rapid-release gelatin sponge (LR-G), and medical-grade gelatin sponge without LPS (MG) (Figure 1A).

## 2. Materials and Methods 

### 2.1. Materials

Two porcine-skin-derived type-A gelatin powders, namely medical-grade gelatin RM-100 and reagent-grade gelatin G2500, were purchased from Jellice Co., Ltd. (Miyagi, Japan) and Sigma-Aldrich Co. LLC. (St. Louis, MO, USA) respectively. LPS from *Escherichia coli* O55 was purchased from Sigma-Aldrich Co. LLC.

### 2.2. Lipopolysaccharide (LPS) Content Measurement

The LPS contents of the medical- and reagent-grade gelatin in Milli-Q water were measured utilizing a ToxinSensor^TM^ Chromogenic LAL Endotoxin Assay Kit (L00350, GenScript USA Inc., Piscataway, NJ, USA) according to the manufacturer’s instructions. 

### 2.3. Preparation of Gelatin Sponges

#### 2.3.1. Preparation of Medical-Grade Gelatin Sponge without LPS (MG)

Medical-grade gelatin powder (100 mg) was dissolved in 10 mL of Milli-Q water at 70 °C. The resultant solution was poured into silicone tubes (5 mm in diameter, 7 cm in height) and stored for 24 h at −30 °C. The tube contents were lyophilized utilizing a DC800 lyophilizer (Yamato Co., Ltd., Tokyo, Japan), then subjected to DHT via vacuum heating utilizing an ETTAS AVO-250NS vacuum dryer (AS ONE, Osaka, Japan) for 24 h at 150 °C with a gauge pressure of −0.1 MPa to obtain the MG. The cylindrical columns of MG were dissected indiscriminately (3 mm in diameter, 2–3 mm in height). The sponges were then saturated with 50 μL of saline prior to the animal experiments.

#### 2.3.2. Preparation of LPS Sustained-Release Gelatin Sponge (LS-G)

Reagent-grade gelatin powder (100 mg) was dissolved in 10 mL of Milli-Q water at 70 °C. The following steps were the same as those for the preparation of MG. The cylindrical columns of LS-G were dissected indiscriminately (3 mm in diameter, 2–3 mm in height). The sponges were saturated with 50 μL of saline prior to the animal experiments.

#### 2.3.3. Preparation of LPS Rapid-Release Gelatin Sponge (LR-G)

The dissected MG were saturated with LPS-containing saline (1.7388 EU/μL) to obtain LR-G (containing 12.42 EU/mg of LPS). All sponges were stored at 4 °C in the dark prior to their use.

### 2.4. Characterization of Sponges

Macroscopic observations were conducted utilizing a Canon A495 camera (CANON Inc., Tokyo, Japan). Field-emission scanning electron microscopy (SEM, S-4800, Hitachi, Tokyo, Japan) was employed to confirm the porous structures of the MG (MG is the base material of LR-G) and LS-G. SEM images were obtained with parameters of 5.0 kV and 10 μA. Attenuated total reflection Fourier transform infrared spectroscopy (IRAffinity-1S, Shimadzu, Kyoto, Japan) was used to confirm the chemical structures of the MG and LS-G. Data preprocessing algorithms were utilized to adjust the baseline measurements and eliminate noise in the spectra via smoothing.

### 2.5. LPS Release Experiments from Sponges

Gelatin sponge samples (1 mg) were placed into 1 mL of sterile saline and shaken at room temperature for 3 days. The LPS levels in the resulting solutions were measured utilizing a ToxinSensor^TM^ Chromogenic LAL Endotoxin Assay Kit (L00350, GenScript USA Inc.) according to the manufacturer’s instruction. 

### 2.6. Implantation of Sponges

All animal experiments were approved by the Animal Experiment Committee of Osaka Dental University and strictly conformed to the guidelines (Approval No. 19-03006). Sprague Dawley rats (male, 8 weeks old) were anesthetized prior to operation utilizing an intraperitoneal injection of a mixture of butorphanol tartrate, midazolam, and medetomidine hydrochloride. In the center of the calvaria of each rat, critical-size defects (9 mm in diameter) was created utilizing a trephine bar (Dentech, Tokyo, Japan). Sterile saline was added occasionally during the procedure to decrease bone damage. The defects were filled with 7 mg of sponge (the LR-G was weighed prior to saturation). In the cases of MG and LS-G, dissected sponges with 50 μL of saline were implanted in the defects. In the case of LR-G, dissected MG sponges saturated with 50 μL of LPS-containing saline were implanted in the defects. A negative control (no-implant) group of rats had only saline added to their defects. The rats were divided into the following groups: 1 = No implant, 2 = MG, 3 = LS-G, and 4 = LR-G. A total of 36 rats were utilized for the experiments (3 rats × 12 groups, including the no-implant control group, for 1, 2, and 3 weeks). At 1, 2, and 3 weeks after implantation, groups of rats were euthanized and the treated calvaria were harvested and fixed with a 4% phosphate-buffered paraformaldehyde solution (FUJIFILM Wako Pure Chemical Co., Osaka, Japan) for further evaluation.

### 2.7. Hematoxylin-Eosin Staining

Four-micrometer-thick non-decalcified frozen sections were obtained from the fixed samples utilizing the Kawamoto method [30]. Thin sections were then processed via hematoxylin-eosin staining. Images were captured utilizing a BZ-9000 digital microscope (Keyence Co., Osaka, Japan). Histomorphometric analysis was conducted to calculate the thickness of tissues above the original bone level in the calvaria defect areas utilizing Adobe Photoshop Elements (Adobe Systems Inc., San Jose, CA, USA) and ImageJ (Image J 1.50i; NIH, Bethesda, MD, USA). This process was conducted as follows: (1) capture images by utilizing the BZ-9000 digital microscope, (2) draw a continuous curve along each outer side of the bone beds from end to end to mark the original bone surface utilizing Adobe Photoshop Elements, and (3) measure the distances from the margin vertexes of tissue to the original bone surfaces marked in (2) utilizing ImageJ.

### 2.8. Lysate LPS-Level Test

All tissue in the defect areas on days seven, 14 and 21 was collected and lysed in sterile saline (2 mL). The LPS levels in the acquired solutions were measured utilizing a ToxinSensor^TM^ Chromogenic LAL Endotoxin Assay Kit (L00350, GenScript USA Inc.) according to the manufacturer’s instructions.

### 2.9. Immunohistochemistry Analysis

Immunostaining was performed to observe inflammatory reactions and cell senescence. The sections obtained by utilizing the Kawamoto method were incubated with a Tris-HCl buffer and blocked with BLOXALL® Endogenous Peroxidase and Alkaline Phosphatase Blocking Solution (SP-6000, Vector Laboratories, Burlingame, CA, USA) at room temperature. Next, incubation with primary antibodies (Anti-COX2/Cyclooxygenase 2 antibody (ab15191, Abcam, Cambridge, MA, USA), Anti P16-INK4A (10883-1-AP, Proteintech, Rosemont, IL, USA), and Anti P21 (10355-1-AP, Proteintech)) was conducted at 4 °C overnight and incubation with the corresponding secondary antibody (Alexa Flour^TM^ 488 goat anti-rabbit immunoglobulin G (H+L); A11034, Thermo Fisher Scientific, Rockford, IL, USA) was conducted for 30 min at room temperature. Next, the sections were mounted with a ProLong^TM^ Gold anti-fade reagent with 4’,6-diamidino-2-phenylindole (DAPI; P36935, Thermo Fisher Scientific). After staining, the sections were observed utilizing a confocal laser microscope (LSM-700, Zeiss Microscopy, Jena, Germany) and the obtained images were analyzed utilizing ImageJ to evaluate the positive staining areas of p16 and p21 according to the following formula:

$$\;\mathrm{Fluorescent}\;\mathrm{staining}\;\mathrm{area}\;\left(\%\right)=\frac{\;p16\;\mathrm{or}\;p21\;\mathrm{positive}\;\mathrm{staining}\;\mathrm{area}\;}{\mathrm{total}\;\mathrm{area}\;\mathrm{of}\;\mathrm{tissue}\;}\times 100\;$$

Three micrographs of each group were used. 

### 2.10. Bone Histomorphometric Analysis Utilizing Soft X-ray Imaging

The fixed samples were evaluated utilizing soft X-ray equipment (SOFTEX: CSMW-2, Softex Co., Ebina, Kanagawa, Japan) operating at 20 kV with 4 mA radiation. The exposure time was 1.0 s. Three rats per group were utilized for bone histomorphometric analysis. The obtained images were analyzed utilizing ImageJ to evaluate the area of new bone growth according to the following formula:

$$\mathrm{Bone}\;\mathrm{area}/\mathrm{total}\;\mathrm{defect}\;\mathrm{area}\;\left(\%\right)=\frac{\mathrm{radiopaque}\;\mathrm{area}}{\mathrm{total}\;\mathrm{area}\;\mathrm{of}\;\mathrm{defect}\;}\times 100.$$

### 2.11. Statistical Analysis

Results are expressed as mean ± standard deviation (SD). One-way analysis of variance (ANOVA) was utilized to compare the mean values between groups. If an ANOVA result was significant, the Tukey-Kramer test was utilized as a post hoc test. All statistical analyses were performed utilizing Prism 8 (GraphPad Software Inc., San Diego, CA, USA). 

## 3. Results

### 3.1. Preparation and Characterization of Sponges

Reagent-grade chemicals have the potential to contain LPS [7]. We first verified LPS contamination in two types of gelatins (reagent-grade and medical-grade gelatin). The reagent-grade gelatin contained 12.42 ± 1.557 EU/mg of LPS and the medical-grade gelatin contained 0.001373 ± 0.0002533 EU/mg of LPS. Remarkably, the LS-G retained its LPS better compared to the intact reagent-grade gelatin and corresponding lyophilized sample (Figure 1B). Although the LR-G contained the same amount of LPS (12.42 EU/mg) as the LS-G, it released all of its LPS within 24 h in saline, whereas the LS-G retained its LPS (Figure 1C and Figure S1).

**Figure 2:**
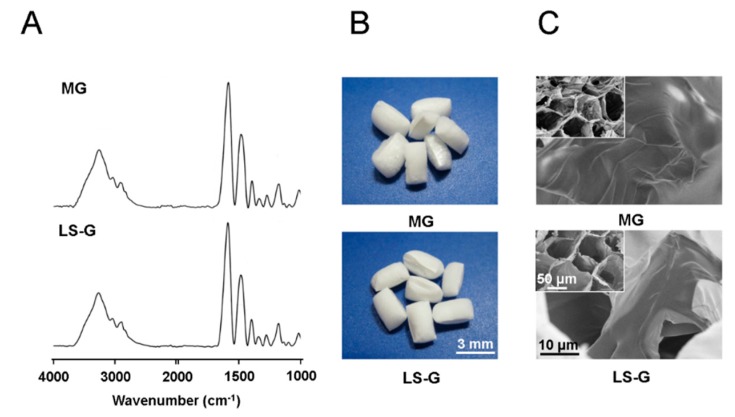
(**A**) Fourier transform infrared (FT-IR) spectra of sponges. (**B**) Macroscopic images of sponges. (**C**) Field-emission scanning electron microscopic (SEM) images of sponges.

Figure 2A presents the Fourier-transform infrared (FT-IR) spectra of MG before adding the LPS-containing saline (for LR-G) and of LS-G. The LPS peak is undetectable for both sponges, but gelatin peaks are visible. Both type of sponges exhibit a spongy morphology with similar pores and smooth surfaces (Figure 2B,C). These results suggest that there were negligible differences between the structures of LS-G and MG before adding the saline or LPS-containing saline prior to implantation into the bone defects.

### 3.2. Inflammatory Reactions of Defects

**Figure 3:**
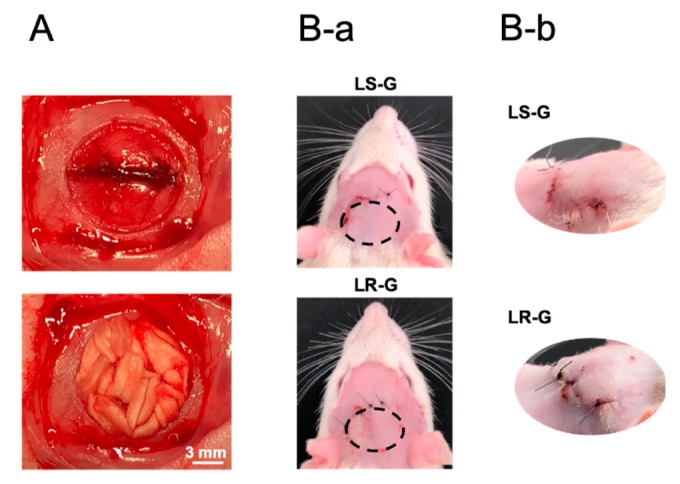
(**A**) Critical-sized bone defects with and without sponges. Fifteen representative pieces of LR-G are presented. (**B-a**) Vertical and (**B-b**) lateral macroscopic views of skin above the surgery site 1 week after surgery.

Figure 3A shows the implanted sponges at the operation. Seven days after the implantation of sponges into defects, no groups showed any obvious necrosis at the incision site and exhibited normal local skin conditions (Figure 3B-a and Figure S2). However, the LR-G group exhibited extraordinary swelling compared to the LS-G group, indicating a stronger inflammatory reaction to the implanted material (Figure 3B-b). The swelling was likely to be attributed to the cellular infiltrate, extracellular matrix (ECM) expansion or the presence of edema. 

**Figure 4:**
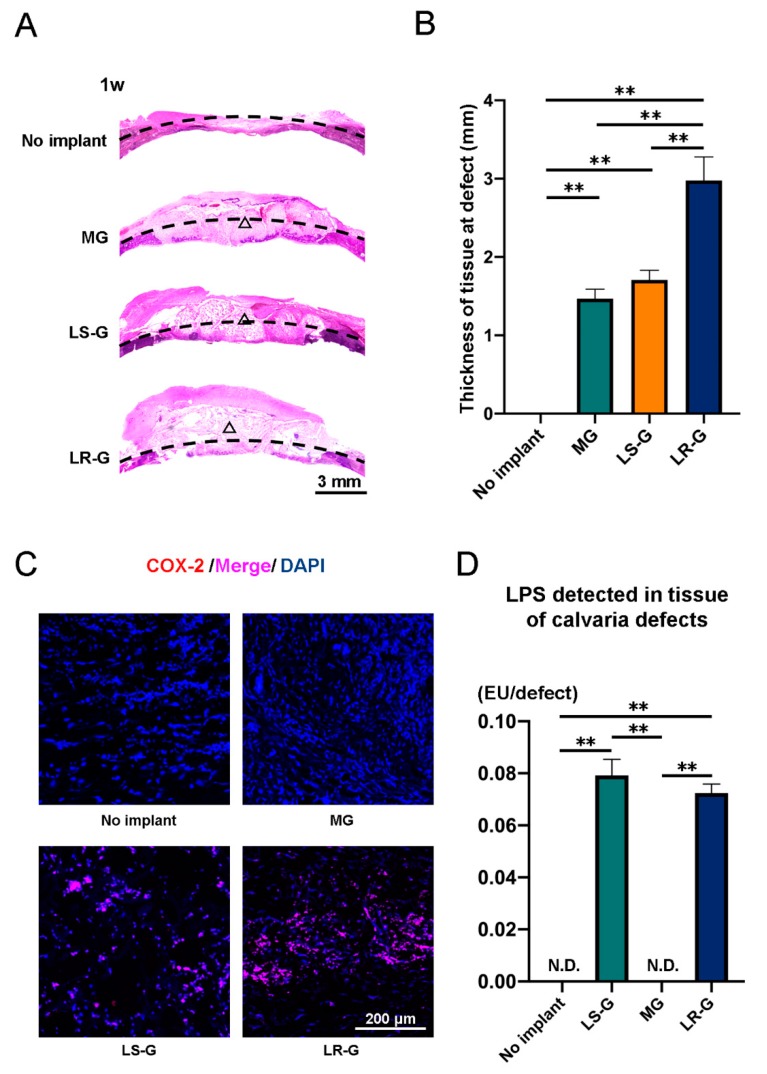
One week after operation: (**A**) Hematoxylin-eosin (H-E) staining and (**B**) quantitative evaluation of tissue thickness at defect sites (△ = gelatin sponge). (**C**) Immunohistochemistry staining with cyclooxygenase 2 (COX-2) and 4′,6-diamidino-2-phenylindole (DAPI). (**D**) LPS levels in lysates from tissue at calvaria defects. Mean with SD (*n* = 3). ** *p* < 0.01: one-way analysis of variance (ANOVA) with Tukey–Kramer test as post hoc test. N.D.: Not detected.

**Figure 5:**
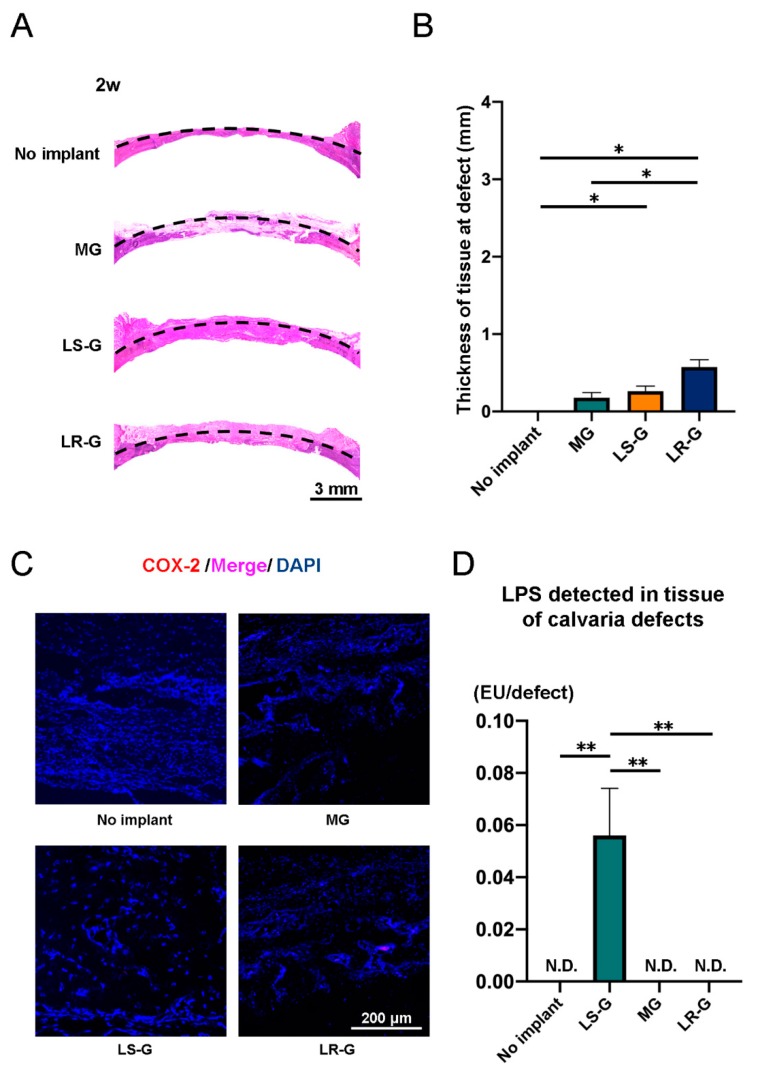
Two weeks after operation: (**A**) H-E staining and (**B**) quantitative evaluation of tissue thickness at defect sites. (**C**) Immunohistochemistry staining with COX-2 and DAPI. (**D**) LPS levels in lysates from tissue at calvaria defects. Mean with SD (*n* = 3). * *p* < 0.05 and ** *p* < 0.01: one-way ANOVA with Tukey–Kramer test as post hoc test. N.D.: Not detected.

**Figure 6:**
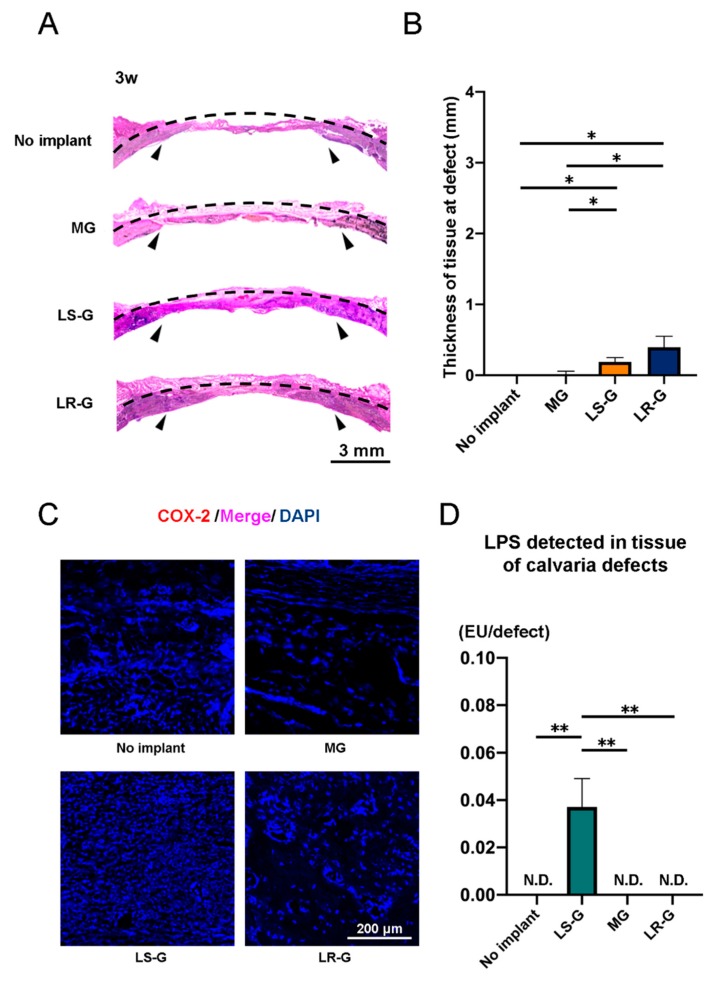
Three weeks after operations: (**A**) H-E staining and (**B**) quantitative evaluation of tissue thickness at defect sites. (**C**) Immunohistochemistry staining utilizing COX-2 and DAPI. (**D**) LPS levels in lysates from tissue in calvaria defects. Mean with SD (*n* = 3). * *p* < 0.05 and ** *p* < 0.01: one-way ANOVA with Tukey–Kramer test as post hoc test. N.D.: Not detected.

To confirm the host reactions at the defect sites, we stained the defects with hematoxylin and eosin (H-E), and cyclooxygenase 2 (COX-2) one, two, and three weeks after surgery (Figure 4A–C, Figure 5A–C, and Figure 6A–C). The LR-G group exhibited thicker tissue at the defect sites compared to the other groups at 1 week (Figure 4A,B). COX-2 is known to be associated with inflammation and the generation of prostaglandin endoperoxide H2 [31]. The expression of COX-2 was strong in the defects treated with the LR-G (Figure 4C). The results of LPS detection for the LS-G and LR-G groups reveal negligible differences in terms of residual LPS in the defects (Figure 4D). These results suggest that the LR-G induced the strongest swelling, but no significant differences in the residual LPS level at one week.

At 2 weeks, the LS-G and LR-G groups showed no significant differences in terms of tissue thickness or COX-2 expression at the defect sites (Figure 5A–C). Residual LPS was only detectable in the defects of the LS-G group (Figure 5D). 

At 3 weeks, the LS-G and LR-G groups exhibited no significant differences in terms of tissue thickness or COX-2 expression at the defect sites (Figure 6A–C). Residual LPS was only detectable in the defects of the LS-G group (Figure 6D). 

### 3.3. Senescent Cells in Defects

**Figure 7:**
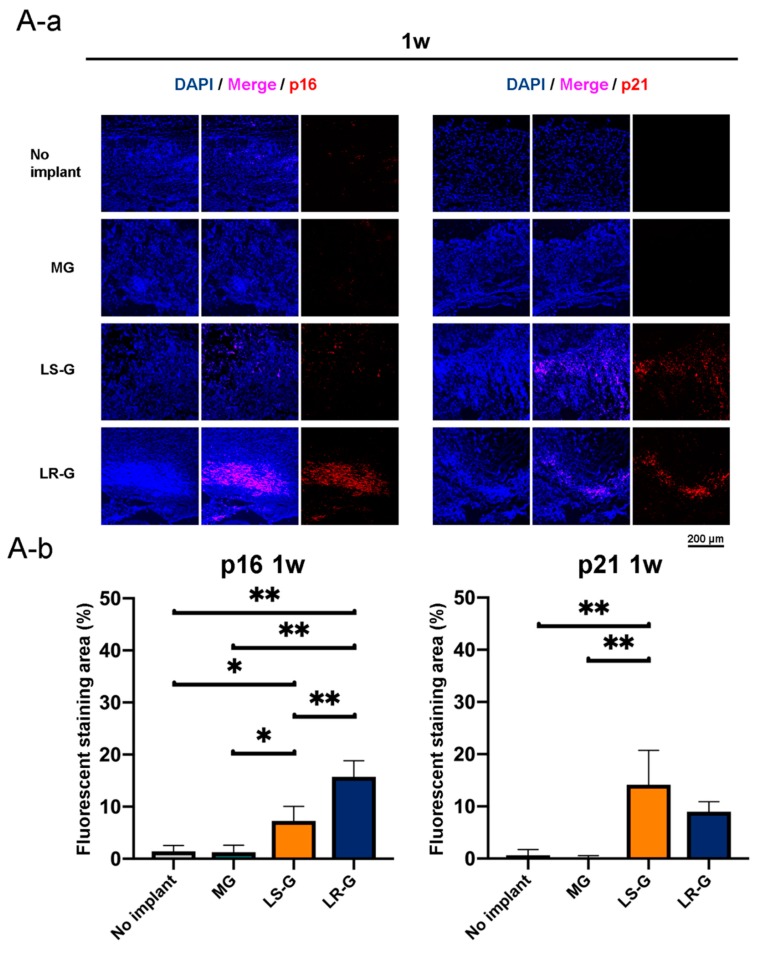

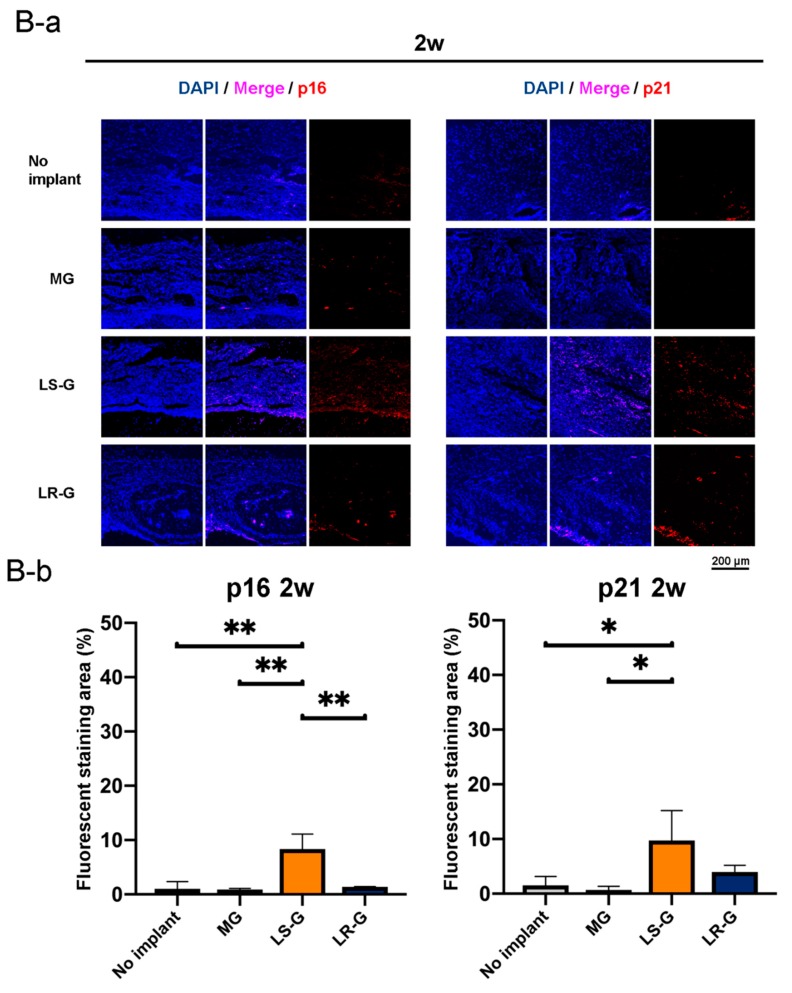

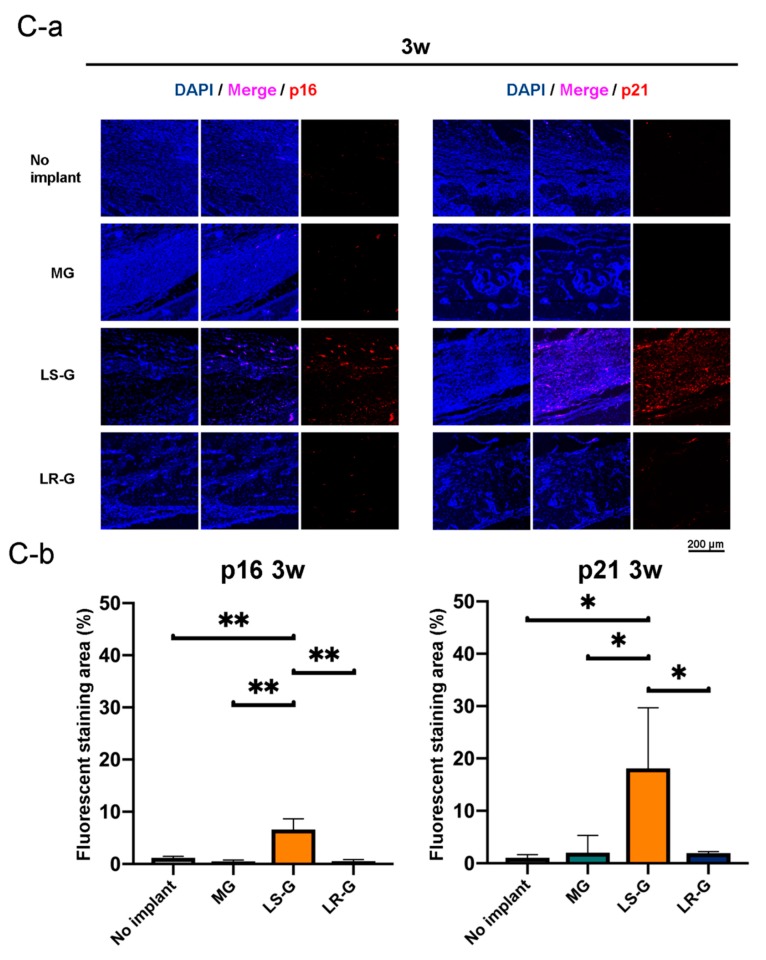
(**A**) One week, (**B**) 2 weeks, and (**C**) 3 weeks after operations: immunohistochemistry staining utilizing DAPI with p16 or p21 (**a**) and the corresponding quantitative data (**b**). Mean with SD (*n* = 3). * *p* < 0.05 and ** *p* < 0.01: one-way ANOVA with Tukey–Kramer test as post hoc test.

Figure 7 presents immunohistochemical staining images and quantitative data regarding the senescent cells in defects 1 to 3 weeks after surgery. The p16 and p21 markers are commonly utilized to distinguish cellular senescence [32,33]. One week after the surgeries, there were p16- and p21-positive cells in the defects treated with both the LR-G and LS-G, but not in the defects treated with the MG or without sponge implantation (Figure 7A). Senescent cells gradually decreased in the defects treated with the LR-G over time, whereas the LS-G retained senescent cells in the defects for up to 21 d (Figure 7B,C). These results indicate that both the LS-G and LR-G similarly induce senescent cells in defects, but with different durations. The implantation of LS-G stimulated senescent cells in the defects for long durations, even without severe inflammation (Figure 6C). 

### 3.4. Bone Formation in Defects

**Figure 8:**
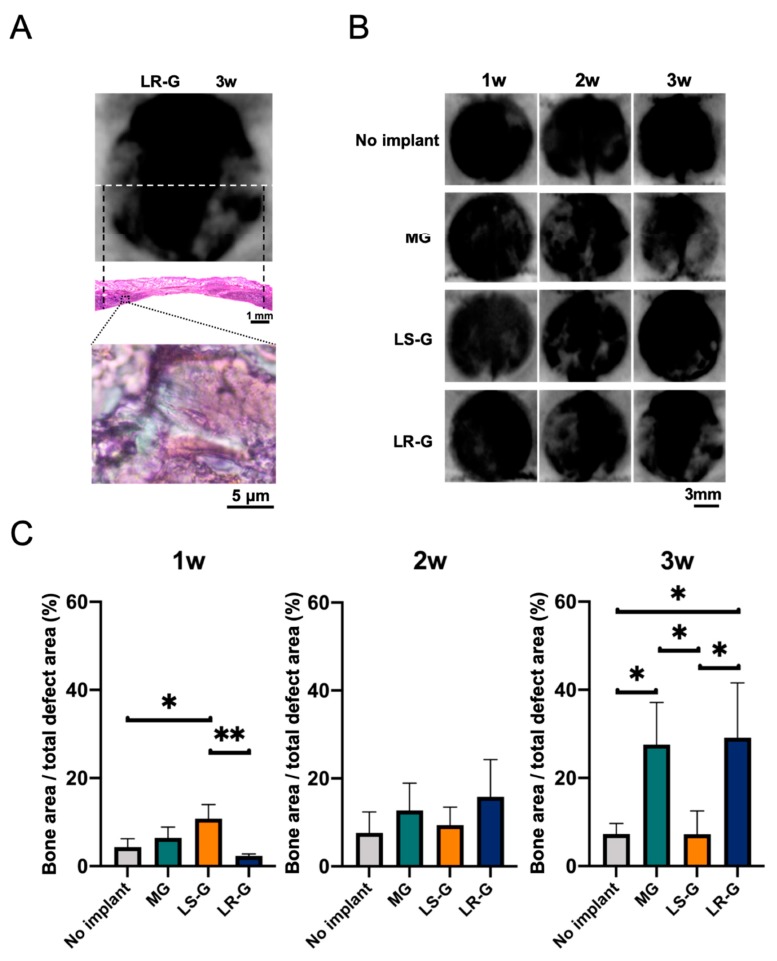
(**A**) Confirmation of bone formation utilizing representative soft X-ray images of the LR-G in defects at 3 weeks and H-E staining results. Upper image: soft x-ray images. White line: cutting line for H-E staining. Lower image: low- and high-magnification defects stained with H-E. (**B**) Radiopaque portion of each defect in the soft X-ray images. (**C**) Morphometric analysis to quantify radiopaque areas (new bone) in defects. Mean with SD (*n* = 3). * *p* < 0.05 and ** *p* < 0.01: one-way ANOVA with Tukey–Kramer test as post hoc test.

Soft X-ray imaging was utilized to evaluate bone formation in early stage in each defect (Figure 8). First, we attempted to determine if radiopacity is a suitable marker for newly formed bone based on H-E staining (Figure 8A). The radiopacity is coincident with the newly formed bone at 3 weeks after the surgeries. There are no obvious increments in radiopacity in the defects treated without sponges for up to 3 weeks. At one week, the defects treated with LS-G exhibited higher radiopacity than those in the other groups, while those treated with LR-G exhibited the lowest radiopacity. At 3 weeks, the defects treated with MG and LR-G exhibited increased radiopacity, but not with LS-G (Figure 8C). These results clearly suggest that the residual duration of LPS in bony defects is likely to alter the course of bone formation. 

## 4. Discussion

Our results demonstrate that different LPS release behaviors not only alter inflammatory reactions, but also cellular senescence and bone formation in critical-sized bone defects in rat calvaria. The sustained release of LPS is coincident with the failure of bone formation for up to 3 weeks. 

Thus far, only a few studies have reported that gelatin can induce inflammation in host cells [34,35]. Similar to one previous study [7], we found that reagent-grade gelatin contains a small amount of LPS (much less than the lethal dose 50 (LD50)) [36]. In this study, contaminated LPS was readily released from intact reagent-grade gelatin when it was dissolved in saline (Figure 1B). Additionally, the LS-G robustly retained its LPS, which consisted of polysaccharides with numerous hydroxyl groups [37]. DHT can enhance the ester bonding between carboxyl and hydroxyl groups [29]. The dehydration condensation of carboxyl in gelatin and hydroxyl groups in LPS can occur following DHT, thereby altering the release behavior of LPS from LR-G and LS-G. Applying DHT to LPS-containing gelatin may be a viable technique for delaying LPS release and transforming the complex into a long-term stimulant for host cells, even in vivo. 

Pore size and porosity alter the bone-forming capabilities of biomaterials [38]. According to SEM observations, MG and LS-G exhibit similar pore sizes before adding an LPS-containing solution or saline. To the best of our knowledge, there has been little research on the relationships between cellular senescence and the pore sizes of biomaterials. Constricting pores leads to DNA damage in cells [39,40], potentially leading to the induction of cellular senescence [13]. However, we could not identify p16- and p21-positive cells in defects treated with the MG. These results indicate that the cellular senescence induced by the LR-G and LS-G must not be caused by intact pores, but by other stimulants in the sponges. 

LPS is believed to enhance the generation of reactive oxygen species (ROS), including H_2_O_2_ and O_2_^−^, resulting in oxidative stress [41,42]. Inflammation caused by the increased production of ROS [43,44] is likely to promote cellular senescence [13]. However, the bone defects 3 weeks after the surgeries in our study exhibited little inflammation at the defect sites (Figure 6). Residual LPS could be found only in defects treated with the LS-G. Repeated [16] or long-term [45] stimulation by LPS causes cellular senescence in vitro. These results may suggest that it is not extracellular reactive oxygen or inflammatory cytokines, but residual LPS that directly promotes cellular senescence.

The LS-G and LR-G contained an equal amount of LPS (28.98 pg per defect). This dose is far less than the LD50 (3 mg/kg) [36]. Previous studies utilized 345 to 17,253 times more LPS compared to our doses in vivo [17,18,46], which apparently hampered bone formation [18,46]. As with these studies, in our study, although LR-G rereleased LPS immediately, bone formation was still delayed for 1 week, which might due to the temporal burst effect (inducing high dose of LPS). Meanwhile, the LS-G only released 1.47% of the amount of LPS released by the LR-G secreting relatively high dose of LPS temporarily. Regardless, the LS-G significantly attenuated bone formation for up to 3 weeks. Guo et al. demonstrated that LPS inhibits osteoblastic differentiation via apoptosis (at 10 ng/mL) [47]. Although we could not exclude the possibility that the low dose of LPS in the LS-G directly inhibited bone formation in the defects, Xu et al. reported that LPS can enhance osteoblastic differentiation at much higher concentration (at 500 ng/mL) [12]. The hampered bone formation might be occurred by different mechanism partially associated with LPS-induced senescent cells.

This study analyzed the effects of LPS release behavior on cellular senescence and bone formation in critical-sized bone defects in rat calvaria. However, further evaluations should be conducted to identify robust relationships between LPS-induced cellular senescence and bone formation. First, it is still unclear how LPS-induced cellular senescence alters bone formation in defects. Second, elucidating the fate and quality of newly formed bones with different types of sponges would be very informative. Third, it is still unclear whether released- or unreleased-LPS from LS-G caused the cellular senescence. We may also need to clarify the differences in LPS between the LS-G and LR-G. 

Overall, our study demonstrated that vacuum heating techniques can be utilized to fabricate two distinct types of gelatin sponges for releasing LPS rapidly or gradually. Such sponges significantly affect inflammatory reactions, cellular senescence, and bone formation in an early stage in critical-size bone defects in rat calvaria. Additionally, these two materials enabled us to determine that the sustained release of LPS attenuates bone formation and increases cellular senescence in vivo. The mechanisms determining how these senescent cells hamper bone formation are still unclear. However, the results suggest that the approaches for preparing different types of materials releasing bacterial components are likely to be helpful for developing disease models that provide an informative insight to design novel biomaterials for bone-regenerative therapy. 

## Supplementary Materials

The following are available online at https://www.mdpi.com/1996-1944/13/1/95/s1, Figure S1: LPS release from LS-G into saline over 3 days, Figure S2: (A) Vertical and (B) lateral macroscopic views of skin above the surgery site 1 week after surgery.
