# Supplementary material for: Releasing Behavior of Lipopolysaccharide from Gelatin Modulates Inflammation, Cellular Senescence, and Bone Formation in Critical-Sized Bone Defects in Rat Calvaria
Source: Materials (Basel). 2019 Dec 23;13(1):95. doi: 10.3390/ma13010095 (PMC6981995; doi:10.3390/ma13010095)
Supplement: Supplementary file 1 [file materials-13-00095-s001.pdf]

materials

MDPI

Article

Releasing Behavior of Lipopolysaccharide from Gelatin Modulates Inflammation, Cellular Senescence, and Bone Formation in Critical-Sized Bone Defects in Rat Calvaria

Jianxin Zhao [1], Yoshitomo Honda [2,*], Tomonari Tanaka [3], Yoshiya Hashimoto [4] and Naoyuki Matsumoto [1]

[1] Department of Orthodontics, Osaka Dental University, 1-5-17, Otemae, Chuo-ku, Osaka 540-0008, Japan; jianxinzhao@hotmail.com (J.Z.); naoyuki@cc.osaka-dent.ac.jp (N.M.)
[2] Institute of Dental Research, Osaka Dental University, 8-1, Kuzuhahanazonocho, Hirakata, Osaka 573-1121, Japan.
[3] Graduate School of Science and Technology, Kyoto Institute of Technology, Matsugasaki, Sakyo-ku, Kyoto 606-8585, Japan; t-tanaka@kit.ac.jp
[4] Department of Biomaterials, Osaka Dental University, 8-1, Kuzuhahanazonocho, Hirakata, Osaka 573-1121, Japan; yoshiya@cc.osaka-dent.ac.jp
* Correspondence: honda-y@cc.osaka-dent.ac.jp; Tel.: +81-72-864-3130

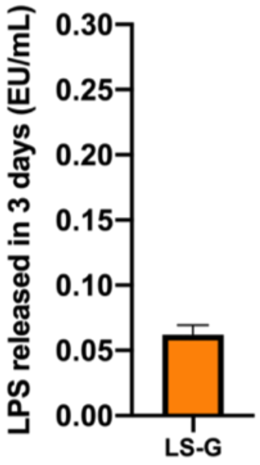

Figure S1. LPS release from LS-G into saline over 3 days.

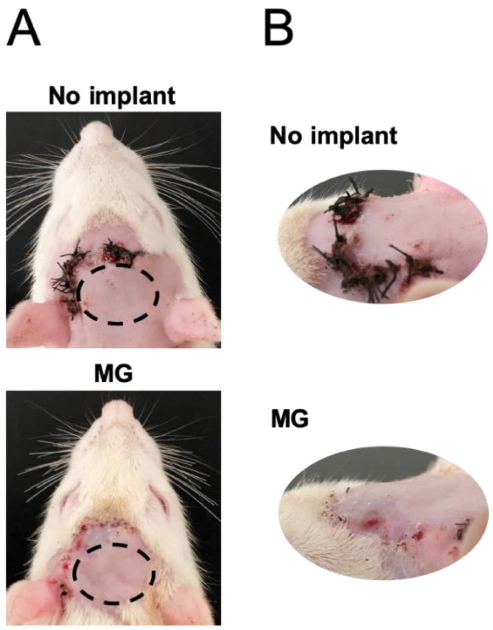

Figure S2. (**A**) Vertical and (**B**) lateral macroscopic views of skin above the surgery site 1 week after surgery.

© 2019 by the authors. Submitted for possible open access publication under the terms and conditions of the Creative Commons Attribution (CC BY) license (http://creativecommons.org/licenses/by/4.0/).
